# Willingness to Adopt Health Information Among Social Question-and-Answer Community Users in China: Cross-sectional Survey Study

**DOI:** 10.2196/27811

**Published:** 2021-05-21

**Authors:** PengFei Li, Lin Xu, Tingting Tang, Xiaoqian Wu, Cheng Huang

**Affiliations:** 1 Medical Informatics College Chongqing Medical University Chongqing China; 2 Medical Data Science Academy Chongqing Medical University Chongqing China; 3 The Children's Hospital of Chongqing Medical University Chongqing China

**Keywords:** health information adoption, social question-and-answer community, structural equation model, Zhihu

## Abstract

**Background:**

COVID-19 has spread around the world and has increased the public’s need for health information in the process. Meanwhile, in the context of lockdowns and other measures for preventing SARS-CoV-2 spread, the internet has surged as a web-based resource for health information. Under these conditions, social question-and-answer communities (SQACs) are playing an increasingly important role in improving public health literacy. There is great theoretical and practical significance in exploring the influencing factors of SQAC users’ willingness to adopt health information.

**Objective:**

The aim of this study was to establish an extended unified theory of acceptance and use of technology model that could analyze the influence factors of SQAC users’ willingness to adopt health information. Particularly, we tried to test the moderating effects that different demographic characteristics had on the variables’ influences.

**Methods:**

This study was conducted by administering a web-based questionnaire survey and analyzing the responses from a final total of 598 valid questionnaires after invalid data were cleaned. By using structural equation modelling, the influencing factors of SQAC users’ willingness to adopt health information were analyzed. The moderating effects of variables were verified via hierarchical regression.

**Results:**

Performance expectation (β=.282; *P*<.001), social influence (β=.238; *P*=.02), and facilitating conditions (β=.279; *P*=.002) positively affected users’ willingness to adopt health information, whereas effort expectancy (*P*=.79) and perceived risk (*P*=.41) had no significant effects. Gender had a significant moderating effect in the structural equation model (*P*<.001).

**Conclusions:**

SQAC users’ willingness to adopt health information was evidently affected by multiple factors, such as performance expectation, social influence, and facilitating conditions. The structural equation model proposed in this study has a good fitting degree and good explanatory power for users’ willingness to adopt health information. Suggestions were provided for SQAC operators and health management agencies based on our research results.

## Introduction

After the outbreak of COVID-19, the Chinese government implemented community isolation measures to control the spread of the epidemic, and because of these measures, the internet became the public’s primary tool for searching for health information. Web-based knowledge-sharing platforms such as Zhihu (Zhihu Inc) and other social question-and-answer communities (SQACs) have been playing an increasingly important role in disseminating health information to the public. By 2019, Zhihu had more than 220 million registered users and had produced more than 28 million questions and 130 million answers. In recent months, the topic of COVID-19 has attracted more than 10,000 followers who had more than 13,000 related questions. Zhihu also has more than 20 million followers in their *Health* section and more than 750,000 health-related posts. We believe that exploring the willingness to adopt health information (WAHI) among users of SQACs could characterize community users’ health information needs and behaviors. We believe that the findings of this study can promote public health literacy by improving the quality and efficiency of SQAC users’ health information searches, adoption, and use.

Health information has flourished as a topic in recent information behavior literature. Many scholars have conducted in-depth research on health information resources [1-3], requesters [3-5], dissemination environments [6,7], and technology [8,9]. Specifically, after the epidemic of COVID-19 began, the research area of health information behavior attracted more attention [10-12]. Health information adoption is the final step in the health information diffusion path; it directly determines the effects of any information dissemination, and because of its significance, many researchers have investigated health information adoption among SQAC users. For instance, Diviani et al [13] found that low health literacy and related skills were negatively related to the ability to evaluate web-based health information and trust such information. Lee et al [14] investigated the level of seeking health-related information on the internet and how health literacy, access to technology, and sociodemographic characteristics impact behaviors related to seeking health-related information. Additionally, Ridout and Campbell [15] reviewed relevant research on young people’s acquisition of health information from social networking sites and found that social networking site–based interventions were highly usable, engaging, and supportive for young people. More and more research findings have determined that further research on the influence factors of health information adoption intentions among SQAC users is necessary and meaningful.

SQACs are public social media platforms in which normal users both search for and share experiences and knowledge on any given topics [16]. These web-based communities have the characteristics of professionalism, interactivity, and open editing [17], and they have high potential influence on health information dissemination. Multimedia Appendix 1 shows a screenshot of the Zhihu website, and each part of the website is annotated in detail. In the existing research on SQACs, the two primary areas of focus have been platform design [18,19] and user behavior [16,17,20]. Jin et al [20] explored patient behaviors related to seeking health care information on SQACs based on dual-process theory and the knowledge adoption model, and Shi et al [21] compared the health information needs of Chinese and American patients with diabetes. The latter authors found that communities from different countries had different attributes. One commonality in extant health information adoption research is that it is grounded in the text information that already exists on social media platforms. There is far less research on SQAC users’ health information behaviors based on users’ own subjective self-reports.

Venkatesh et al [22] proposed the unified theory of acceptance and use of technology (UTAUT) by summarizing the advantages and disadvantages of other theoretical models in the field of technology acceptance. The core factors of the UTAUT include performance expectation (PE), effort expectancy (EE), social influence (SI), and facilitating conditions (FCs); gender, age, experience, and the voluntariness of use were used as moderators in the model. Due to the native characteristics of the UTAUT model, it has a stronger affinity and adaptability for most acceptance behavior research. Many scholars have embraced the UTAUT as a new theoretical blueprint in technology acceptance behavior research and have incorporated it into a vigorous acceptance behavior research stream. Bawack and Kamdjoug [23] conducted an empirical study on the acceptance of hospital information systems in limited-income countries based on the UTAUT. In a similar study based on the adjusted UTAUT, Nunes et al [24] found that gender, age, and other individual characteristics were moderating factors of users’ willingness to adopt health applications. Abdelhamid [25] extended the UTAUT model and found that higher autonomy positively affected patients’ willingness to exchange health information. Zhou et al [26] studied the mobile health (mHealth) information retrieval behavior of South African college students based on the UTAUT and found that perceived usefulness was the main influencing factor of their willingness to accept health information exchange. Liu et al [27,28] established a taxonomy of clinical decision support interventions based on the UTAUT model and tried to explore how patient care can be improved through clinical decision support. The UTAUT model has been used widely in the research area of users’ acceptance behaviors. However, few scholars have used the UTAUT model to study SQAC users' WAHI. The goal of this study was to establish an effective model for analyzing SQAC users’ WAHI based on the UTAUT model and to identify the influencing factors of users’ WAHI.

### Hypotheses and Modeling

#### PE Factor

PE is defined as the degree to which an individual believes that using a system will help them attain gains in job performance [22]. In the environment of SQACs, PE refers to users’ perceptions of the benefits of adopting health information. Researchers measure PE with the following five structural variables: perceived usefulness, extrinsic motivation, job fit, relative advantage, and outcome expectations [29]. Based on the environment of SQACs, we proposed four additional PE items [29-31] on our questionnaire scale, as follows: (1) “the health information on Zhihu can play an important role in understanding and solving health-related problems,” (2) “making full use of the health information on Zhihu can help me better solve some problems,” (3) “the health information on Zhihu has helped me or others around me improve our health,” and (4) “the health information on Zhihu has a high reference value for my health decision-making.” In general, researchers have shown that PE has positive effects on acceptance [32,33]. On the basis of these findings, we proposed the following research hypothesis: PE positively affects SQAC users’ WAHI (H1).

#### EE Factor

In the UTAUT model, EE is defined as the degree of ease associated with the use of a system [22]. In this study, it refers to a user’s perception of the difficulty of adopting relevant health information in SQACs and applying such information to practice. The following three constructs capture the concept of EE: the perceived ease of use, complexity, and ease of use [22]. Cimperman et al [34] and Adenuga et al [35] found that EE was a positive variable in models, and we proposed the following two scale items based on the previously mentioned studies [22,36] for our questionnaire: (1) “the health information on Zhihu is easy to understand” and (2) “the health recommendations on Zhihu are usually easier to implement.” Users are more likely to adopt health information that is easy to understand and implement, and on the basis of these findings, we proposed the following hypothesis: EE positively affects SQAC users’ WAHI (H2).

#### SI Factor

Venkatesh et al [22] defined SI as the degree to which an individual perceives that it is important for others to believe that they should use a new system, and SI can be represented by a subjective norm, social factors, and an image. The differences in health levels between other people before and after adopting health information will affect individuals’ willingness to accept of health information. On the basis of previous research findings [36,37] and the three dimensions of SI measurement proposed by Venkatesh et al [22], we proposed the following three scale items: (1) “there are other people available to look up or solve health-related problems on Zhihu,” (2) “other people around me have pushed health information from Zhihu to me,” and (3) “Zhihu’s image makes me feel that it will help me understand or solve health-related problems.” On the basis of the research finding that SI has a positive effect on acceptance [38-40], we proposed the following hypothesis: SI positively affects SQAC users’ WAHI (H3).

#### FCs Factor

FCs refer to the perceived (organizational, societal, etc) convenience of or support for adopting something new, such as a new technology [22]. In this study, we investigated the FCs of perceived health information convenience and support among SQAC users. Health information support in an SQAC environment mainly comprises the following three aspects: (1) community users’ assistance with understanding relevant health information, (2) the convenience and ease of obtaining relevant health information from users, and (3) individual users’ own knowledge and experiences. On the basis of these features [36], we proposed three questionnaire items, as follows: (1) “I have the resources, hardware and knowledge reserve to effectively use the health information on Zhihu,” (2) “I can get help from others when I encounter problems while browsing and consulting health information on Zhihu,” and (3) “seeking health information on Zhihu is one of the common ways for me to understand and solve health-related problems.” Garavand et al [41] found that FCs had a positive effect on major students’ adoption of mHealth apps. On the basis of these research findings, we proposed the following hypothesis: FCs positively affect SQAC users’ WAHI (H4).

#### Perceived Risk

Perceived risk (PR) is one of the most important and widely used concepts in psychology, economics, and other fields. At present, the widely used measurement dimensions of PR include economic risk, time risk, information security risk, and health risk. PR is also one of the important determinants of health information adoption; the higher the perceived risk, the lower the willingness to adopt such information. A study [42] of Chinese patients’ intention to adopt mHealth services showed that PR negatively affects respondents’ trust and willingness to accept mHealth services. Although the UTAUT model integrates many variables from different classical models, it does not include the impact of PR on users' willingness to accept technology. Therefore, we decided to extend the PR variable in the model. Physiological risk, psychological risk, and time risk are the main risks for SQAC users’ WAHI available on Zhihu. On the basis of the above findings, we proposed the following three items for our questionnaire scale: (1) “taking advice from relevant health information on Zhihu could cause physiological harm,” (2) “taking advice from the health information on Zhihu could cause some psychological pressure,” and (3) “the health information on Zhihu could be a useless waste of my time.” Furthermore, we proposed the following hypothesis: PR negatively affects SQAC users’ WAHI (H5).

#### Moderating Variables

Moderating variables such as gender, age, and the voluntariness of use play a very important role in the original UTAUT model [22]. The original UTAUT model is a widely used measurement model. However, when measuring and explaining a phenomenon with the aid of the UTAUT model, the variables in the model must be adjusted based on objective facts. When users decide whether to adopt certain health information, they are faced with high health costs, which are directly related to their own or other people's health. Compared to the intention to adopt information technology, the influence of the voluntariness of use variable on SQAC users’ WAHI is less important. Therefore, the voluntariness of use variable was removed from the model. Demographic characteristics may have different levels of significance in terms of their effects on the WAHI. Haluza et al [43] found that sociodemographic attributes, including gender influence, not only affect private the web-based habits of users but might also affect the acceptance of health technologies and their professional use in a clinical setting. Tarver et al [44] found that age is one of the significant social-economic influencing factors of web-based health information communication. Irizarry et al [45] proposed that age and education level strongly influence patient users’ interests and their ability to use patient portals. Based on existing relevant theories, we selected gender, age, and education level as the three moderating variables in the model and proposed the following hypotheses: gender has a significant moderating effect on the influence of independent variables for adoption intention (H6), age has a significant moderating effect on the influence of independent variables for adoption intention (H7), and educational level has a significant moderating effect on the influence of independent variables for adoption intention (H8).

#### Model

**Figure 1:**
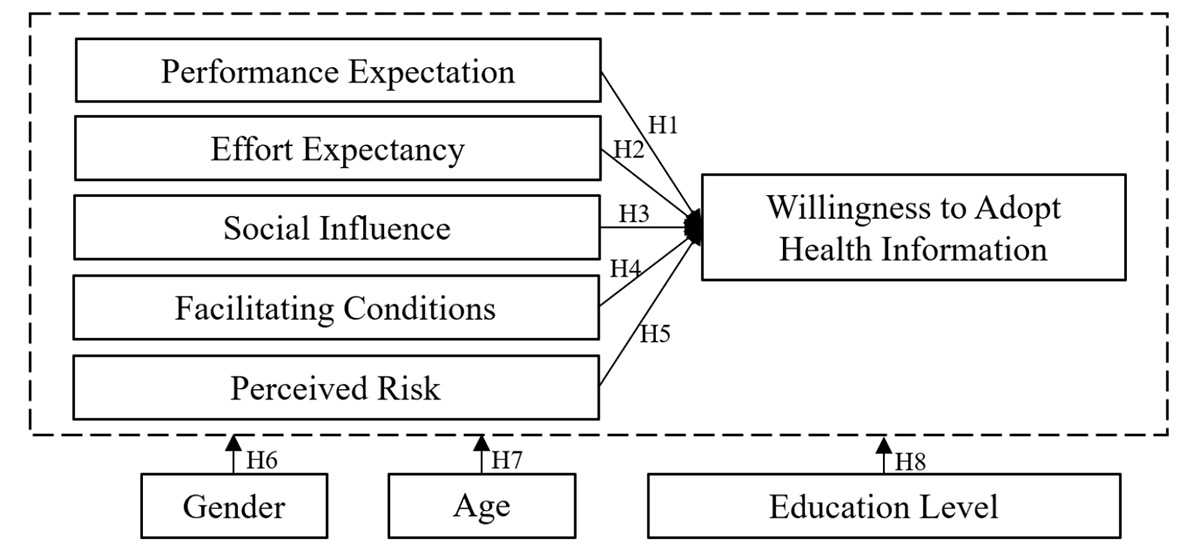
The model of social question-and-answer community users’ willingness to adopt health information.

Based on the structural characteristics of SQACs and SQAC users’ adoption of health information, we reset the moderating variables of the UTAUT model and incorporated PR variables into the UTAUT model to construct the final model of SQAC users’ WAHI. We present the model in Figure 1.

## Methods

### Participant Selection and Data Collection

We selected users who exhibited health information behaviors (health information browsing, commenting, searching, and other relevant behaviors) on Zhihu as the respondent sample for this study. On the basis of our literature review findings, we designed a web-based questionnaire survey scale. The scale was comprised of the items we previously outlined, and it was used to measure 19 indicators of 6 variables related to the WAHI among users of SQACs. All scale items were rated on 5-point Likert scales. We selected 22 postgraduates as presurvey participants to test the availability and quality of the questionnaire. Based on the presurvey results of the questionnaire, we deleted one of the index items for the variable EE (ie, “it often takes more time to retrieve health information in Zhihu”). After adjusting the questionnaire content and structure, the final scale was chosen via expert discussion. We included screening items in the final demographic indicators section of the questionnaire to ensure that all of the data we used came specifically from the Zhihu user group. We administered the web-based survey over 14 days between June 5 and 19, 2020, on the web-based questionnaire platform Wenjuanxing (Liepin Holdings Limited). The questionnaire was distributed by Zhihu users’ forwarding the questionnaire link via WeChat (Tencent Holdings Limited). Before the survey process could advance, we presented the content of this study and required respondents to confirm their informed consent for participating further in this study. In total, data from 921 participants’ were collected in this study. After filtering out the data of the nonusers of Zhihu and data with missing values and obvious errors, valid data from 598 participants were obtained, with an effective rate of 64.93%. A detailed list of questions can be found in Multimedia Appendix 2.

### Statistical Analysis

We used Microsoft Excel for data cleaning and preprocessing before the data analysis. The statistical analysis tools we used in this study mainly included SPSS version 24.0 (IBM Corporation), Analysis of Moment Structures (AMOS) version 24.0 (IBM Corporation), and Process macro version 3.3 for SPSS [46]. Considering that the age distribution of Zhihu users was relatively concentrated, to avoid having a sample size of 0 for certain age groups and to increase the rate of unnecessary systematic errors, we grouped samples by age (20-year increments). With the structural equation model, we calculated the path coefficients between variables to verify the hypotheses. With Process, we conducted hierarchical regression to verify whether the moderating effects of each moderating variable in the model were significant. We set a *P* value of less than .05 as statistically significant.

### Quality Control

Composite reliability and the Cronbach α are the most commonly used indicators of questionnaire reliability. As shown in Table 1, the Cronbach α and composite reliability values for each variable, except those for EE, were greater than .7, and the overall Cronbach α value for the questionnaire was .917. These results indicated the good reliability of the questionnaire that we developed for this study [47]. The validity testing of questionnaires entails testing two components—content and structure validity. As our model and the index expression were verified by others many times, the questionnaire had high content validity. Structural validity consists of convergent and discriminant validities. Convergent validity requires the factor loading and average variance extraction (AVE) values for each index item to exceed .5. As shown in Table 1, all of the variables in this study exceeded this threshold, except for EE. Discriminant validity requires the correlation coefficient of each variable to be less than the square root of the AVE value of the variable itself, and Table 2 reflects that the questionnaire passed the discriminant validity test.

**Table 1:** The factor load, Cronbach α, average variance extraction (AVE), and composite reliability (CR) values of each variable.

Variables and indices			Factor load		Cronbach α		AVE		CR
**PE^a^**					.881		.636		.883
	PE1	.804							
	PE2	.819							
	PE3	.824							
	PE4	.787							
**EE^b^**					.450		.450		.621
	EE1	.657							
	EE3	.685							
**SI^c^**					.774		.531		.773
	SI1	.745							
	SI2	.688							
	SI3	.752							
**FC^d^**					.760		.520		.764
	FC1	.783							
	FC2	.662							
	FC3	.714							
**PR^e^**					.819		.603		.819
	PR1	.789							
	PR2	.812							
	PR3	.725							
**WAHI^f^**					.852		.596		.854
	WAHI1	.716							
	WAHI2	.821							
	WAHI3	.704							
	WAHI4	.838							

^a^PE: performance expectation.

^b^EE: effort expectancy

^c^SI: social influence.

^d^FC: facilitating condition.

^e^PR: perceived risk.

^f^WAHI: willingness to adopt health information.

**Table 2:** Discriminant validity matrix.^a^

Variables	Performance expectation	Effort expectancy	Social influence	Facilitating condition	Perceived risk	WAHI^b^
Performance expectation	.797	N/A^c^	N/A	N/A	N/A	N/A
Effort expectancy	.535	.671	N/A	N/A	N/A	N/A
Social influence	.589	.507	.729	N/A	N/A	N/A
Facilitating condition	.609	.449	.590	.721	N/A	N/A
Perceived risk	.038	.289	.160	.144	.777	N/A
WAHI	.607	.444	.553	.576	.125	.772
AVE^d^	.636	.450	.531	.520	.603	.596

^a^The diagonal of the matrix is the square root of AVE of the corresponding variable.

^b^WAHI: willingness to adopt health information.

^c^N/A: not applicable.

^d^AVE: average variance extraction.

## Results

### Demographic Characteristics

The demographic characteristics of the participants in this study are shown in Table 3. Of the 598 respondents, 69.90% (n=419) were female and 94.15% (n=563) were aged 19 to 38 years. Just over three-quarters of the survey respondents (477/598, 79.77%) had an undergraduate or higher education.

**Table 3:** Statistical description of the sample.

Variables and categories			Value, n (%)
**Gender**			
	Male	179 (29.93)	
	Female	419 (69.90)	
**Age (years)**			
	≤18	21 (3.51)	
	19-38	563 (94.15)	
	39-58	14 (2.34)	
**Education**			
	Senior high school and below	7 (1.17)	
	Junior college	10 (1.67)	
	Undergraduate	477 (79.77)	
	Master and above	104 (17.39)	

### Model Test

We completed the path verification of the model with AMOS 24.0 and SPSS 24.0, and Table 4 presents the model fitting findings. The model fit indices that we calculated were chi-square to df ratios, the root mean square error of approximation (RMSEA), the normed fit index, the relative fit index, the incremental fit index, the Tucker-Lewis index, and the cumulative fit index. Conventionally, model fit is considered good when the chi-square to df ratio is <3, the RMSEA is <0.08, and the normed fit index , relative fit index , incremental fit index, Tucker-Lewis index, and cumulative fit index are >0.9 [48,49].

**Table 4:** Model fitting.

Indices and values		Standard value	Fitting
**Chi-square to df ratio (χ^2^/*df*)**			
	2.954	<5	Acceptable
	2.954	<3	Ideal
**RMSEA^a^**			
	0.057	<0.08	Acceptable
	0.057	<0.05	Ideal
**Normed fit index**			
	0.929	>0.9	Ideal
**Relative fit index**			
	0.912	>0.9	Ideal
**Incremental fit index**			
	0.952	>0.9	Ideal
**Tucker-Lewis index**			
	0.940	>0.9	Ideal
**Cumulative fit index**			
	0.952	>0.9	Ideal

^a^RMSEA: root mean square error of approximation.

The structural equation model and the path coefficients are shown in Table 5. Paths “EE to WAHI” (*P*=.79) and “PR to WAHI” (*P*=.41) were not significant, whereas paths “PE to WAHI” (β=.282; *P*<.001), “SI to WAHI” (β=.238; *P*=.02), and “FC to WAHI” (β=.279; *P*=.002) were significant. Our findings supported H1, H3, and H4 but failed to support H2 and H5.

**Table 5:** Path test of the structural equation.

Hypotheses	Path	Unstandardized estimates	Standardized estimates (SE^a^)	Critical ratio	*P* value	Results
H1	PE^b^ to WAHI^c^	.280	.282 (.084)	3.314	<.001	Accept
H2	EE^d^ to WAHI	.036	.027 (.141)	0.256	.79	Reject
H3	SI^e^ to WAHI	.224	.238 (.098)	2.296	.02	Accept
H4	FC^f^ to WAHI	.262	.279 (.085)	3.080	.002	Accept
H5	PR^g^ to WAHI	.030	.032 (.036)	0.825	.41	Reject

^a^SE: standard error.

^b^PE: performance expectation.

^c^WAHI: willingness to adopt health information.

^d^EE: effort expectancy.

^e^SI: social influence.

^f^FC: facilitating condition.

^g^PR: perceived risk.

### Moderating Effect Test

**Figure 2:**
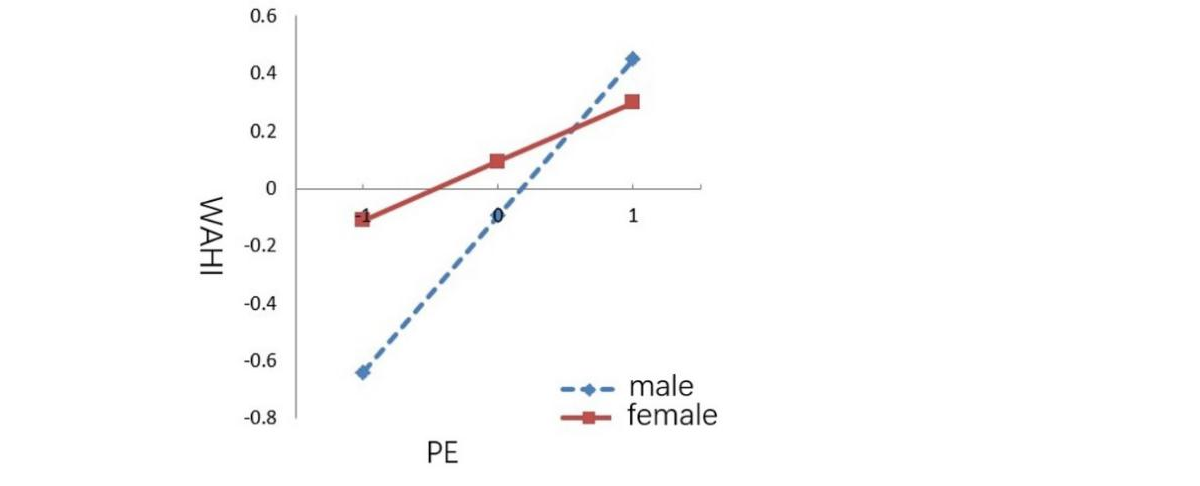
The moderating effect of gender on the "PE to WAHI" path. PE: performance expectation; WAHI: willingness to adopt health information.

With Process macro version 3.3 for SPSS, we completed the testing of the moderating effects of the moderating variables in the model, and we present the specific significance of moderating effects in Table 6. The results in Table 6 indicate that gender significantly moderated the effect that PE had on SQAC users’ WAHI. This supported H6. However, H7 and H8 were not supported. Table 7 shows the model parameters from before and after we incorporated gender as a moderating variable. With regard to model 1, Table 7 presents the standardized coefficients of each variable and the corresponding R^2^ and *F* test values for when the moderating variables were not included. With regard to model 2, Table 7 reflects the parameter changes that occurred after we introduced gender as a moderating variable. Compared to model 1, model 2’s explanatory power for SQAC users’ WAHI improved after we incorporated the different moderating variables. Figure 2 shows a visual representation of the moderating effects in this study. The slope of Figure 2 indicates the effect that PE has on SQAC users’ WAHI. A larger slope means that the model is more sensitive to the WAHI. We found that the slope of the male sample was considerably larger than that of the female sample (Figure 2). Therefore, we believe that the male group had more obvious fluctuations than the female group.

**Table 6:** The significance of moderating effects.

Path	Gender	Age	Education level
PE^a^ to WAHI^b^	√^c^		
SI^d^ to WAHI			
FC^e^ to WAHI			

^a^PE: performance expectation.

^b^WAHI: willingness to adopt health information.

^c^The moderating effect was significant at the .05 level.

^d^SI: social influence.

^e^FC: facilitating condition.

**Table 7:** Hierarchical regression test of moderating effects.

Index	Model 1^a^				Model 2^b^		
	B	*t* test (*df*)	*P* value	B		*t* test (*df*)	*P* value
Performance expectation	0.331	8.126 (597)	<.001	0.375		8.123 (597)	<.001
Social influence	0.210	5.250 (597)	<.001	0.190		5.029 (597)	<.001
Facilitating conditions	0.251	6.171 (597)	<.001	0.249		6.179 (597)	<.001
Gender	N/A^c^	N/A	N/A	0.094		1.913 (597)	<.001
Interaction item^d^	N/A	N/A	N/A	−0.170		−2.390 (597)	.02

^a^Model 1 had an R^2^ value of 0.461 (*P*<.001) and an *F* test value (*F*_3,594_) of 169.643.

^b^Model 2 had an R^2^ value of 0.470 (*P*<.001) and an *F* test value (*F*_5,592_) of 104.804.

^c^N/A: not applicable.

^d^The interaction item for the gender and performance expectation.

## Discussion

### Principal Findings

Since the outbreak of COVID-19, the role of SQACs in the public’s access to health information has grown in prominence. This study was an analysis of the influencing factors of SQAC users' WAHI during the COVID-19 pandemic. We conducted measurements by using a questionnaire that comprised items grounded in the UTAUT and its individual components. Based on our results, PE (*P*<.001) and SI (*P*=.02) had significant positive effects on the WAHI. PE reflects users’ expectations for improved health if they adopt health information, and the WAHI increases in a community if the community users believe that the information is helpful to them. However, in contrast with PE, SI affected WAHI through the people around the users. Improved health among individuals who adopt health information will encourage SQAC users to adopt the health information that is recommended by these individuals. Abdelhamid [25] found that perceived usefulness and SI were the most important factors affecting health information exchange between patients, and our findings were consistent with that conclusion.

Under the premises of compliance and legality, SQAC operators can make full use of the traces of users in SQACs (eg, browsing content, page stay times, likes, collections, content, and the theme of private messages). With the help of this kind of information, SQAC operators could enhance the strength of relationships among different users who pay attention to the same health topics. With the epidemic of COVID-19, people's health concerns have become more focused. This provides a rare opportunity for SQAC operators to improve users’ relationship strength. Furthermore, actively guiding users to share their understanding of and experience with health information is another effective method for improving users’ PEs and SI. By using these methods, we can further enhance users’ WAHI and users’ health levels.

FCs positively affected SQAC users’ WAHI. FCs are an integration of users’ peripheral auxiliary functions in the process of adopting health information. They are formed based on individuals’ levels of comprehension, the convenience of retrieving information from platforms, and other users’ help in understanding health information. When the effectiveness of this auxiliary role improves, the process of SQAC users’ adoption of health information becomes smoother, and users’ confidence in adopting health information increases. Through this virtuous cyclical process, health information is continuously shared, exchanged, and adopted, and improving the health information retrieval mechanism can increase the quality of the health information retrieved.

EE (*P*=.79) and PR (*P*=.41) had no significant effects on SQAC users’ WAHI. We believe that it was the interaction of many factors that led to this result. The native environment and characteristics of the original UTAUT could explain why EE had no significant effects on WAHI. Compared to the cost of users' adoption of information technology or information systems in the study conducted by Venkatesh et al [22], during the establishment of the original UTAUT model, the cost of SQAC users' adoption of health information was lower. To some extent, if the cost of health information adoption is low, the significance level of EE will also be low. However, risk comes from the unknown. Sudhakar et al [50] found that lower education levels were significantly related to lower health literacy. For most SQAC users, higher education means higher information literacy. In this study, irrespective of the total number of SQAC users or the sample size, users’ demographic characteristics reflected a higher educational level, which is important for understanding and using health information. Coughlin et al [51] found that persons with limited health literacy are less likely to use patient web portals. We believe that SQAC users who adopt health information and have lower educational levels face more difficulties and risks than those with higher educational levels. However, there could also be deeper reasons for why EE and PR did not significantly affect SQAC users' WAHI. Our inconclusive results require us to conduct more in-depth and detailed research.

As shown in Tables 6 and 7, the moderating effect of gender on path “PE to WAHI” (*P*=.02) was significant, and Figure 2 shows that the WAHI among men was more sensitive to PE than the WAHI among women. Baumann et al [52] found that gender had different moderating effects on the influencing factors of web users’ health information search behaviors. Through empirical research, Ek [53] found that men received far less informal health information from family members, friends, and colleagues than women. Therefore, the PEs of men are more effective in increasing their WAHI than the PEs of women. SQAC operators should pay more attention to the moderating role of gender in the “PE to WAHI” path when attempting to identify optimal health information dissemination schemes. Different coping strategies should be implemented for users of different genders. However, we can effectively improve the WAHI among SQAC users by differentiating the information that they receive based on user gender.

### Contributions and Limitations

In this study, we proposed a model of the WAHI among users of the Zhihu SQAC that was based on the modification of the native UTAUT, and we found good explanatory power for our model. We also analyzed the different influencing factors of the WAHI among Zhihu users. PE, SI, and FCs were the primary influencing factors, and the effect of PE differed according to gender. We proposed several suggestions and measures that can be implemented based on our research findings in this study.

Although we strove to be rigorous, there were still several limitations in this study. First, this was a questionnaire survey based on the subjective cognition of SQAC users; thus, we could not avoid the interference of various subjective factors associated with self-reporting. Second, this was a cross-sectional study, and as such, it was impossible to observe changes in SQAC users’ WAHI over time. Third, although we ensured that the sample was as representative as possible, there were still some inevitable systematic errors. Fourth, although SQACs are gradually becoming an indispensable platform that users can use to obtain health information, most users are still using search engines and other methods to obtain such information, and we did not adequately explain the interactions among these different sources of health information and users’ willingness to adopt such information. Fifth, the model established in this study is a limited extension of the UTAUT model, which cannot cover all of the influencing factors of the dependent variables. Other variables such as information quality, trust, and medical experience will be modeled and studied as the focus in follow-up research. In addition, there were still several limitations in our choices for variable indices. After considering the efficiency of this study, we excluded some indicators that we subjectively considered unimportant, but whether the inclusion of these indicators would enhance the explanatory power of the model remains to be further studied. Finally, we only attempted to identify the moderating effects of demographic characteristics. Therefore, only gender, age, and education level were selected for verification. Whether other demographic indicators have a significant impact needs to be further verified. In spite of the above limitations, the conclusions and suggestions of this study can be used as references by relevant health management agencies.

### Conclusions

We constructed a UTAUT-based model to explain the WAHI among users of the Zhihu SQAC during the COVID-19 pandemic. We tested our hypotheses by using data from a survey (which we administered on the internet) that we analyzed via structural equation modelling. The results showed that PE, SI, and FCs had positive effects on SQAC users’ WAHI; EE and PR did not affect users’ WAHI. We also found that gender (*P*=.02) had a significant moderating effect in the model. We hold the opinion that enhancing the strength of user relationships and improving users' experiences with SQAC platforms are the most useful methods for improving the WAHI among users of SQACs. In addition, we need to encourage all users to improve their health information literacy. Although there are limitations in this study, SQAC operators, researchers, and policy makers can refer to our results to guide policy decisions.

## Appendix

Multimedia Appendix 1 Notes on Zhihu (Zhihu Inc).

Multimedia Appendix 2 Questionnaire on social question-and-answer community users' willingness to share health information.

## Abbreviations

AMOS: Analysis of Moment Structures
AVE: average variance extraction
EE: effort expectancy
FC: facilitating condition
mHealth: mobile health
PE: performance expectation
PR: perceived risk
RMSEA: root mean square error of approximation
SI: social influence
SQAC: social question-and-answer community
UTAUT: unified theory of acceptance and use of technology
WAHI: willingness to adopt health information
