# Supplementary figures and images for: Willingness to Adopt Health Information Among Social Question-and-Answer Community Users in China: Cross-sectional Survey Study
Source: J Med Internet Res. 2021 May 21;23(5):e27811. doi: 10.2196/27811 (PMC8143873; doi:10.2196/27811)

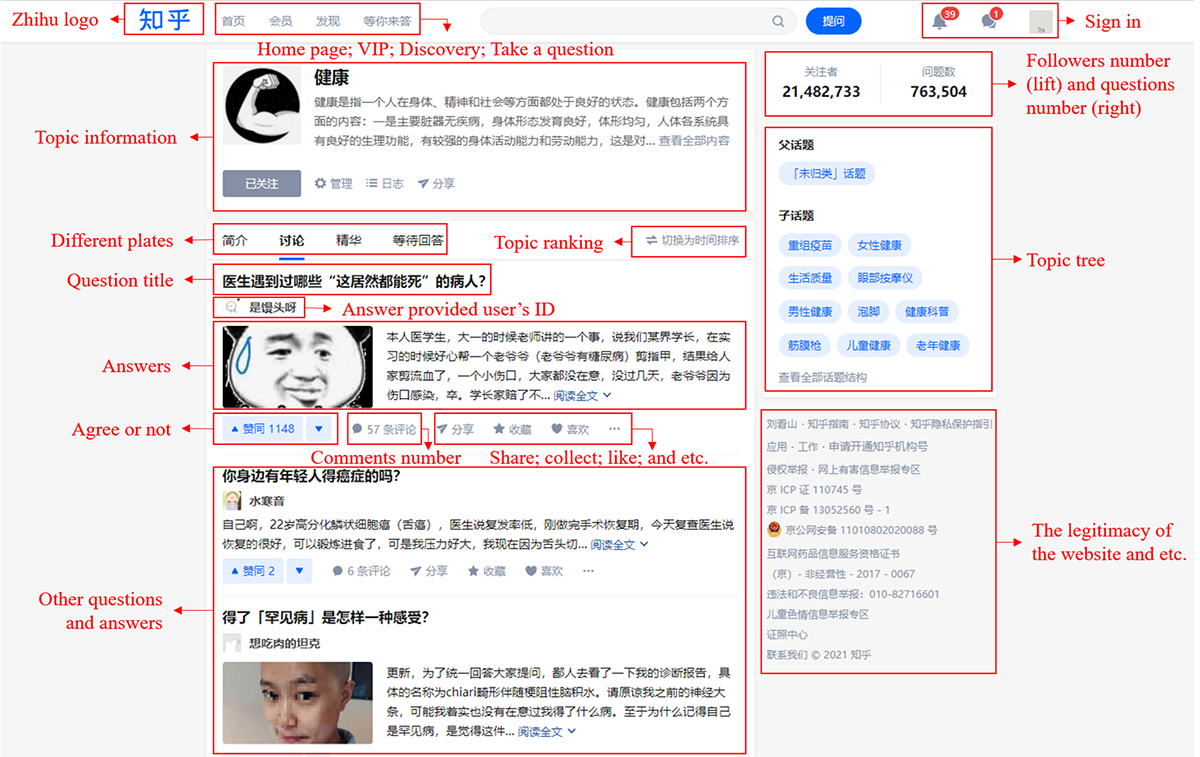

Supplement: Multimedia Appendix 1 [file jmir_v23i5e27811_app1.png]
