# Supplementary material for: Willingness to Adopt Health Information Among Social Question-and-Answer Community Users in China: Cross-sectional Survey Study
Source: J Med Internet Res. 2021 May 21;23(5):e27811. doi: 10.2196/27811 (PMC8143873; doi:10.2196/27811)
Supplement: Multimedia Appendix 2 [file jmir_v23i5e27811_app2.docx]

No._____________

**Questionnaire on the willingness of social Question-and-answer community users to share health information**

Dear Sir/Madam：

Good days!

This is a questionnaire about Zhihu users' willingness to share health information (such as hair loss, staying up late, information, experience and knowledge of COVID-19, etc.). If you have ever used The Zhihu question-and-answer community, we would be grateful if you could use a few minutes to complete the following questionnaire. The research group promises that all your personal answers will only be used for academic research. Please feel free to fill in.

Your serious answer is of great significance to this study. Please fill it out truthfully. Thank you again for your cooperation!

Instructions:

1 Please do not fill in or mention your name in the questionnaire;

2 There is no right or wrong answer, just check it according to your own situation;

3 Please fill in the blanks with your answers at the horizontal line;

4 Please use "√" to check the corresponding option that matches your actual situation.

**Personal Information**

1. gender

①male ②female

2. Age

①≤18 ②19~38 ③39~58 ④59~68

⑤69 and above

3 education

①senior High school and Below ②junior college

③undergraduate ④master and above

4 Background of majors

①medical science or related majors ②non-medical related majors

5 profession

①students

②the government personnel

③professional technical personnel

④business and service personnel

⑤Agricultural production personnel

⑥Production and transportation equipment operators and related personnel

⑦soldier

⑧the others

6 Have you ever used Zhihu

①YES

②NO（Stop answering）

7 The way you come into contact with Zhihu is（Multiple choice）

①Search engine（such as google, Baidu, etc.）

②WeChat ③Microblog

④TV ⑤APP Store

⑥through friends

⑦others：________

8 The health information (such as information, experience and knowledge about pneumonia epidemic, hair loss, staying up late, diet, health maintenance, etc.) you have done on Zhihu include（Multiple choice）

①publish health-related information (including asking questions, answering questions, Posting articles or ideas, etc.).

②comment on health-related information (20 words or more).

③Search for health-related information.

④Click a like for health-related information.

⑤share or forward health-related information (e.g. to WeChat, Microblog, etc.).

⑥Browse for health-related information.

⑦Collect health-related information.

⑧others：________

9 Please evaluate your health level (including physical, mental and social health)

①poor ②medium ③good ④Excellent

**Survey on the willingness to adopt health information on Zhihu**

Please tick "√" in the serial number corresponding to your situation according to your understanding and usage habits.

1: Not at all. → 5: It fits perfectly.

| Questions | Score | | | | |
| --- | --- | --- | --- | --- | --- |
|  | 1 | 2 | 3 | 4 | 5 |
| PE1: The health information in Zhihu has a great effect on understanding and solving related health problems. |  |  |  |  |  |
| PE2: Making full use of the health information in Zhihu can help me solve some problems better. |  |  |  |  |  |
| PE3: Using the health information in Zhihu can improve the health status of myself or others around me. |  |  |  |  |  |
| PE4: The health information in Zhihu can be used as a reference for health decision-making. |  |  |  |  |  |
|  | | | | | |
| EE1: The health information in Zhihu is easy to understand. |  |  |  |  |  |
| EE2: The recommendations made by the health information in Zhihu are usually easier to implement. |  |  |  |  |  |
|  | | | | | |
| SI1: There are other people around to access health information through Zhihu. |  |  |  |  |  |
| SI2: Other people have pushed me health information from Zhihu. |  |  |  |  |  |
| SI3: Zhihu's public image makes me think it is very professional in solving health problems. |  |  |  |  |  |
|  | | | | | |
| FC1: I can effectively use the health-related information resources and knowledge reserves in Zhihu. |  |  |  |  |  |
| FC2: If you have any problems when looking up relevant health information in Zhihu, you can find other friends to help. |  |  |  |  |  |
| FC3: It is one of the common ways for me to understand or solve health-related problems by consulting relevant health information in Zhihu. |  |  |  |  |  |
|  | | | | | |
| PR1: There are certain risks in adopting the health information in Zhihu, which may cause physiological harm. |  |  |  |  |  |
| PR2: There will be some psychological pressure to adopt the health information in Zhihu. |  |  |  |  |  |
| PR3: The relevant health information in Zhihu may waste my time because it is useless. |  |  |  |  |  |
|  | | | | | |
| WAHI1: I would like to praise or support the relevant health information identified in Zhihu. |  |  |  |  |  |
| WAHI2: I am willing to share or forward the beneficial health information in Zhihu to others. |  |  |  |  |  |
| WAHI3: I am willing to restrict my behavior according to the correct health information content in Zhihu. |  |  |  |  |  |
| WAHI4: I am willing to recommend Zhihu Q&A community platform to others based on my own health experience. |  |  |  |  |  |

**The questionnaires are all over. Thank you again for your cooperation！**
